# Influence of fine particle content in debris flows on alluvial fan morphology

**DOI:** 10.1038/s41598-022-24397-x

**Published:** 2022-12-16

**Authors:** Tzu-Yin Kasha Chen, Chi-Yao Hung, Jared Mullenbach, Kimberly Hill

**Affiliations:** 1 Department of Civil Engineering and Hydrotech Research Institute, National Taiwan University, Taipei, 10617 Taiwan; 2 Department of Civil, Environmental, and Geo-Engineering, University of Minnesota, Minneapolis, MN 55455 USA; 3 St. Anthony Falls Laboratory, University of Minnesota, Minneapolis, MN 55414 USA; 4 Department of Soil and Water Conservation, National Chung Hsing University, Taichung, 402 Taiwan; 5 Wood Engineering, Reno, NV 89521 USA

**Keywords:** Natural hazards, Geomorphology, Phase transitions and critical phenomena

## Abstract

Alluvial fans are large-scale depositional structures commonly found at the base of mountain ranges. They are relatively soil-rich compared to the rocky terrains, or catchment areas, from which their material originates. When frequented by debris flows (massive, muddy, rocky flows) they contribute significantly to local hazards as they carry focused, collisional, fast-moving materials across alluvial fans, unpredictable in size, speed, and direction. We research how fine particle content in debris flows correlates with directional changes, i.e., debris flow avulsions. Toward this, we analyzed field data from two neighboring alluvial fans in the White Mountains (California, USA) that exhibit dramatically different topographies despite their proximity and associated similar long-term climates. Informed by these measurements, we performed long-term and incremental alluvial fan experiments built by debris flows with systematically-varied fine particle content. We found that (1) decreasing fine particle content increases the variability of fan slopes and associated channelization dynamics, and (2) for all mixtures longer-term continuous alluvial fan experiments form more complex surface channelizations than repeated flows for the same total time, indicating the importance of *both* particle sizes and timescales on alluvial fan surface morphology.

## Introduction

Alluvial fans are large-scale conical structures that often form in chains (or bajadas^1^) at the base of mountainous regions worldwide: in wetter warmer climates^2–6^, in periglacial climates^7,8^, and in arid environments (where they have been most commonly studied)^1,9–12^. They are primarily comprised of abraded colluvium carried by repeated flows of streams and/or debris flows from steeper catchment areas. Because of associated break-up processes (from abrasion to comminution), fans contain relatively workable regolith compared to the bedrock in their catchments, making the fans particularly suitable for human infrastructure in mountainous areas. Yet, many of these fans were built by debris flows: unpredictable and destructive flows of boulders, gravel, and mud, motivating significant study of similar future events.

**Figure 1:**
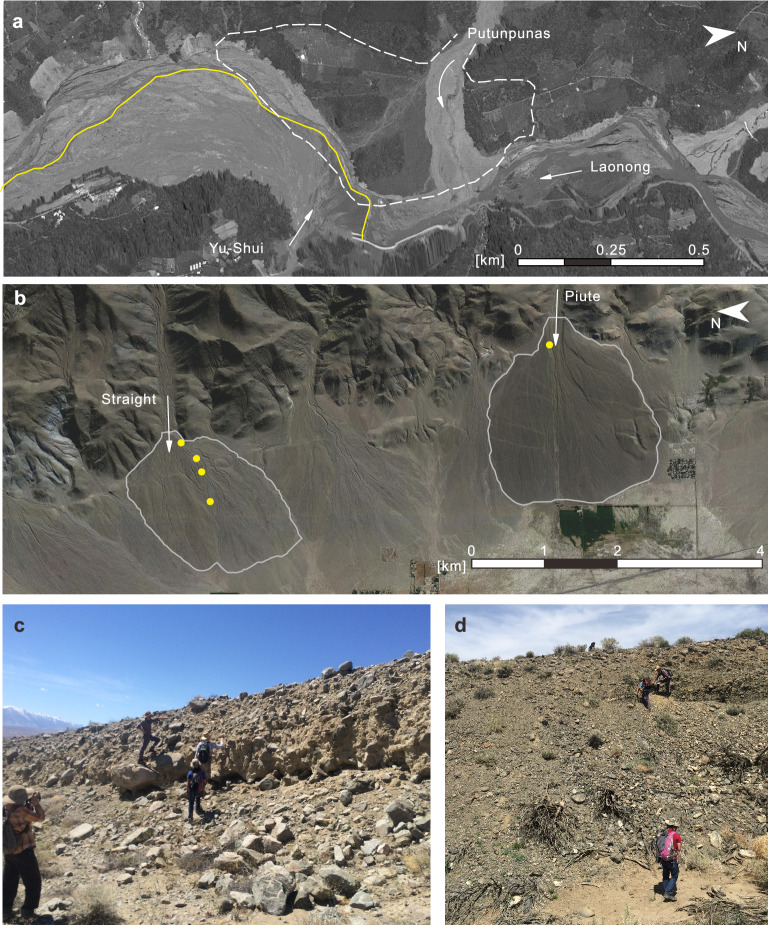
Examples of debris-flow dominated alluvial fans in the field, from (**a**) Laonong River Valley, Taiwan and (**b**–**d**) Owen’s Valley, White Mountains, California, US. (**a**) Laonong river’s satellite image in August, 2021. (1) white lines indicate the DEM-determined Pu-tun-pu-nas fan boundary in February 2020; (2) yellow lines(solid/dash) indicate the DEM-determined Yu-Shui fan boundary in August 2021 (**b**). (**b**) Owens Valley satellite image, September 2019. White lines denote DEM-determined fan boundaries for Straight and Piute. Yellow dots indicate sampling locations. (**c**,**d**), photos with (researchers for scale) on the channel levees in (**c**) straight and (**d**) Piute.

Typically, debris flows travel from the steeper catchment are through relatively narrow canyonlands and into a wider expanse at the fan apex, after which they often channelize the more shallow-sloped fan and deposit. The dynamics of the flows vary with many factors including: grain size distributions, water content, and local slopes. All of these factors are governed by much longer-term dynamics from geomorphological processes to local hydrology and climate. Subsequently, debris flows and their subsequent depositional structures can vary substantially. For example, in wetter tropical regions whose lithology is dominated by sedimentary rock (as in Taiwan, e.g., Fig. 1a) debris flows appear more muddy, at first glance similar to sheet flows, though still suspending and then dispersing gravel, rocks, and massive boulders across much of the fan in single-flow events. In drier areas, single debris-flow events appear rockier and fluid-starved at their fronts (i.e., “snouts”) and are often restricted to a single channel over any particular flow event^13^. Sometimes even several consecutive debris flow events deposit over a relatively narrow fan region^14–16^. Still, among the deposits, certain commonalities define what the scientific community associates with debris-flow dominated fans: large boulders scattered across fan surfaces; relatively high catchment slopes; stream cuts and levees that reveal poorly-sorted thickly-layered deposits, and depositional “lobes”, tongue-shaped depositional structures^17–19^.

Many efforts have been successful at associating properties of catchment regions and alluvial fan structure with likelihood of past or future debris flows and their magnitudes^20–22^. For example, Melton^23^ found a distinguishing measure of debris-flow (rather than stream-flow) dominated fans in Arizona to be what he called the *ruggedness* of the catchment area ($R=H/\sqrt{A}$; *H* and *A* represent the maximum vertical relief and the planview area, respectively). Jackson et al.^20^ (in the Canadian Rockies) and Marchi et al.^21,22^ (in the Italian Alps) found high magnitudes of the Melton number and also slope correlated with debris-flow dominated fans. Recently research been directed toward relating variability in flow *contents* and particular dynamics—specifically *avulsion behaviors*—to distinctions in *depositional structures*^15,16,24–28^. Yet significant questions remain regarding avulsion controls whether it be local rock materials, hydrology, and/or climate forcing.

While significant attention has been paid to grain-size distributions of larger particles^29,30^ relatively little attention has been paid to finer particle grain size distributions in the field (i.e., particles < 2 mm in diameter). However, recent work has demonstrated importance of fine particle content in particle-fluid materials in general and in-channel debris flow dynamics in particular. For example, debris flow experiments at laboratory scale^31–33^ and larger flumes^34,35^ have demonstrated that in-channel debris-flow behaviors, particle sorting and mobility vary significantly with fine particle content. Related multi-scale computational and experimental studies of multi-scale particle-fluid systems^36,37^ have demonstrated the distinct role of fine particle content in mediating system-scale behaviors by modifying larger particle-particle interactions. Motivated by these results, herein we investigate how fine particle content may be related to differences in down-slope avulsion controls using preliminary field observations and systematic laboratory experiments.

## Field-scale studies: particle properties and channel avulsion signatures

To test for evidence of the influence of fine particle content on field-scale avulsion behaviors on alluvial fans, we consider similarities and differences of two fans on the west side of the White Mountains (California, USA) (Fig. 1b–d): Straight and Piute Fans. Evidence of a predominance of debris flows in these fans include: large boulders that litter both surfaces, toe-deposits and levees, and stream cuts that reveal poorly sorted thickly layered deposits (Fig. 1c,d). Additionally, their relatively high slopes and catchment area roughness numbers suggest that these fans were primarily built by debris flows (Supplementary Table 1). Their proximity to one another suggests they developed under similar climatic conditions.

Yet even with these similarities and their proximity, the two fans exhibit substantial differences. From a distance, even their coloration differs, likely due to different mineralogical constituents^38^ (Supplementary Fig. 1). Upon closer investigation, these fans differ in what we might call their *channelization complexity*. Straight fan is riddled with intersecting overlaid channels up to 2 to 4 m deep, while Piute Fan has relatively few surface channels, and they are considerably deeper (Fig. 1c,d). Several samples (Fig. 1b, Supplementary Fig. 2, Tables 2, 3) from the active channel levees on Straight Fan indicate that recent flows down Straight fan deposited, roughly, an even distribution of sand, silt, and clay with insignificant variation down-channel. In contrast, the sample we collected from near Piute’s fan apex and observations of qualitatively similar deposit size distributions downslope indicates a much higher concentration of silt than that of clay or sand.

Though the Piute sampling is limited to one location, we were inspired by the distinct differences from all samples from Straight Fan as well as differences in the lithologies of the two fans^38^. The minerology of the catchments are associated with differences in particle strength and, subsequently, mechanical weathering rates. Based on these observations and measurements, we hypothesize that apparent differences in the lithology and associated fine particle content could indeed influence system-wide behaviors as found by Man et al.^36,37^ in dramatically different boundary conditions. On fans, we suspect the fine particles may affect both the rheology of the flows as well as erosion rates, thereby affecting the balance between down-fan avulsions and in-channel persistence. These considerations motivated more systematic investigations made possible by laboratory basin-scale physical experiments.

## Laboratory experiments

**Figure 2:**
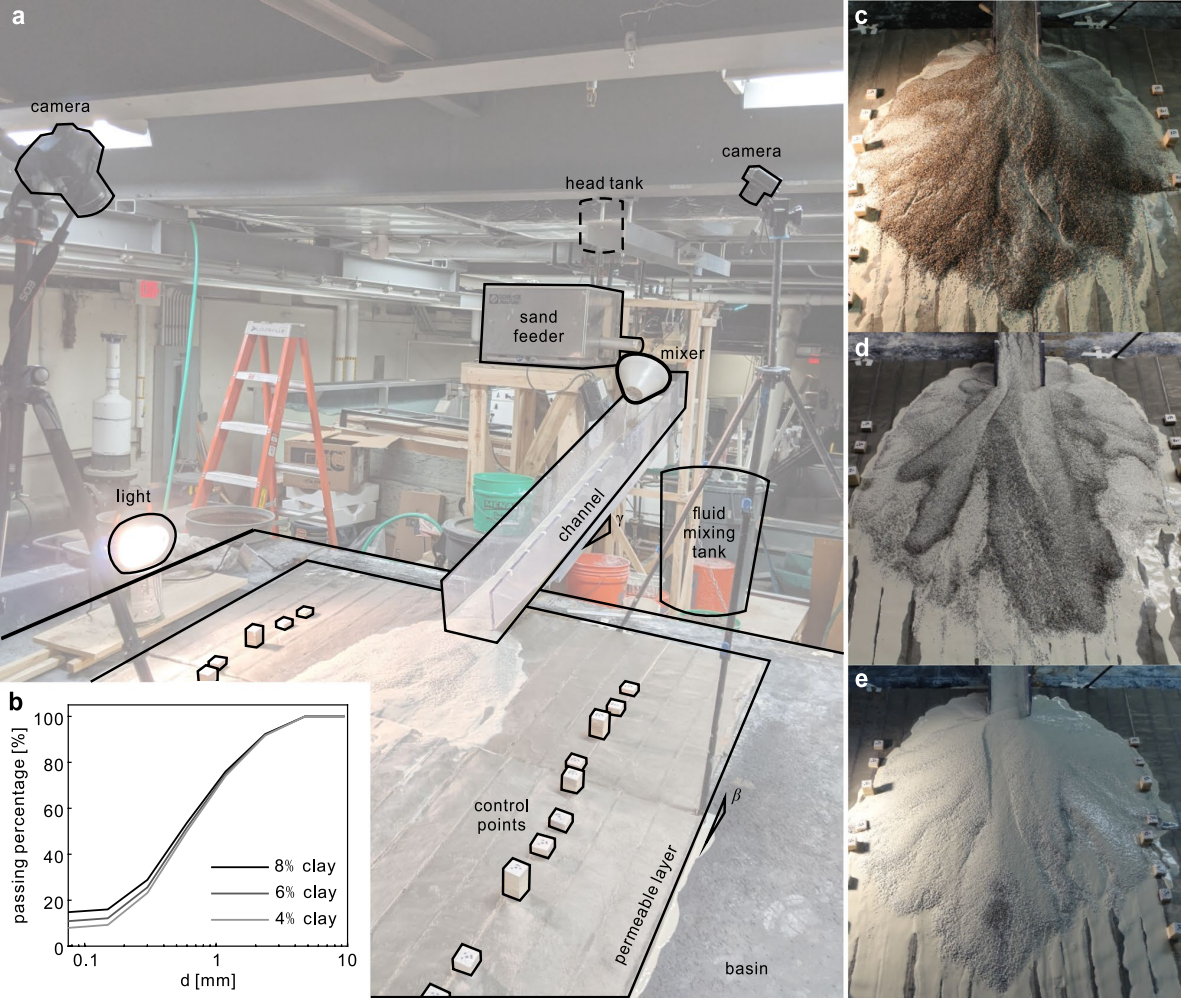
(**a**) Laboratory set-up and (**b**) grain size distribution of our three mixtures (data presented in Supplementary Table 5). (**c–e**) Experimental alluvial fans resulting from continuous 15-min releases with: (**c**) (sand:clay:water) = (50:4:46) by weight; (**d**) (sand:clay:water) = (52:6:42) by weight; (**e**) (sand:clay:water) = (48:8:44) by weight.

We designed large-scale experiments to mimic the structure of the canyon-basin system by using a narrow channel that fed into a wide basin with a permeable surface (Fig. 2a). During each experiment, we continuously mixed and discharged clay/water/flocculent mixtures into a funnel mixer, into which we simultaneously input sand at a steady rate, so that they entered the channel well-mixed at the upstream end and flowed down the channel into the basin. Given the size of our smallest large particles (sand) we made the decision to use clay rather than silt as our experimental fines. We discuss this more in the final sections.

We performed twelve experiments (summarized in Supplementary Table 4) using three different mixtures varying primarily in clay fraction from 4 to 8% (Fig. 2b, Supplementary Table 5). Relatively small variations in water and sand made the experiments run more smoothly; our longer term goals are to understand these effects as well, though beyond the scope of our present focus of the importance of clay differences on experimental fan-scale behaviors. In suspensions, clay particles tend to interact with one another, so the clay-fluid rheology is non-Newtonian (Supplementary Fig. 3, Table 6). We comment on how this is important for the “large” (sand-sized) particle scale interactions in the “Quantitative analyses and discussion” section.

**Figure 3:**
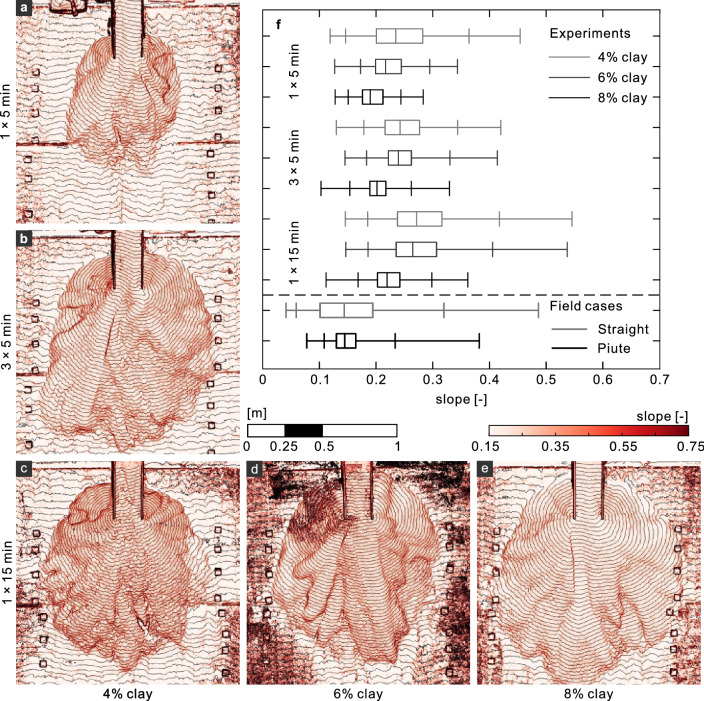
(**a–e**) Elevation contours and slope maps of experimental fans. Contour line spacing represents 5 mm elevation changes. (**a–c**) shows results for runs of constant clay content performed for different discharge timescales: (**a**) one 5-min flow, (**b**) three successive 5-minute flows (**a**) one 15-min continuous flow. (**c–e**) results for runs of one 15-min flow and clay contents of (**c**) 4%, (**d**) 6%, and (**e**) 8%. (More in Supplementary Figs. 3, 4). (**f**) Boxplot for the slope distribution of the fan surface in 9 representative experiments and the 2 field cases we studied. The boxplot displays the dataset based on the 1st, 5th, 25th, 50th, 75th, 95th, and 99th percentiles (Supplementary Table 10).

We initiated two distinct experiments for each mixture over the cleaned permeable surface of the sloped basin. For one, we discharged the mixture continuously for 15 min (Fig. 2c–e), and, for the other, we discharged the mixture continuously for only 5 min (e.g., Fig. 3a). After each 5-min experiment, we performed two additional 5-min experiments over the previous fan without cleaning down to the previous bed. (e.g., Fig. 3b). Since our primary data came from photogrammetry after the deposition was halted, this resulted in a total of twelve fan surfaces (Supplementary Table 7).

We note several commonalities from one run to the next, independent of mixture content, basal conditions, and depositional time (apparent in Supplementary Videos, Tables 8, 9). In all cases we observed the dynamics evolve over time from an initial, roughly radially-symmetric depositional stage that established a relatively uniform erodible bed to a break from symmetric to discrete self-channelizing flows that frequently avulsed primarily by one of two processes. In one process, a more frequently-referenced down-channel blockage^16,28,39^, led to sudden channel abandonment (e.g. Supplementary Video V1, 1:07–1:13). In the other process, an apparent up-channel dominated process, erosional stresses on one channel bank gradually moved the channel sideways resembling channel-bank migration (e.g. Supplementary Video V1, 1:13–1:20 and 1:29–1:35). During the latter phase we observed typical debris flows features including deposition of the flow into discrete lobes, segregation of the “larger particles” (sand) outward onto levees and depositional lobe edges. After deposition was complete, natural coloration helped distinguish levees from in-channel deposit, more difficult to observe during flows, perhaps because of the small experimental scales (e.g., Fig. 2e).

There were also notable differences based on: boundary conditions, flow mixtures, and what might be called “timescales”. For the flows initiated over the cleaned permeable bed, the flow deposited in relatively radially symmetric layers over the base (and independently of the timescale of the well-known “roll waves”^40^, ongoing in the channel). In contrast, the shorter runs we initiated over previously-deposited 5-min flows (Runs 2,3, 6, 7, and 10, 11) appeared at first to follow the partially drained channels, i.e., asymmetrically. Shortly after this initial stage, the flow reset the channels before proceeding to re-channelization stage, avulsion, and channel migration. Increasing clay content changed the apparent time scales of channelization and avulsions (e.g., Supplementary Videos V1, V3, V5, Fig. 3c–e). Our high clay-content flows avulsed more slowly, giving rise to relatively few channels and lobes compared to their low-clay counterparts. Our low clay-content flows more frequently avulsed prior to reaching the fan edge and did so quickly, giving rise to more channels and a bumpier final deposition morphology. Also, the higher-clay flows created wider channels than the lower-clay flows. The difference between a 15 min deposition formed from continuous flows from that formed from repeated shorter flows was also evident in fan morphology, relatively independent of mixture. A fan built over a continuous (15 min) flow event produced a more continuous channelization evolution than one built from the same material over three successive (5 min) flow events. For the latter, each time we terminated the flow, fluid drained from the fan so that restarting the flow first served to re-wet the fan surface, partially resetting the channels (Compare Supplementary Videos V1, V2), giving rise to a final fan surface with fewer lobes and channels (compare Fig. 3b,c), apparently less relief over the fan surface.

## Quantitative analyses and discussion

**Figure 4:**
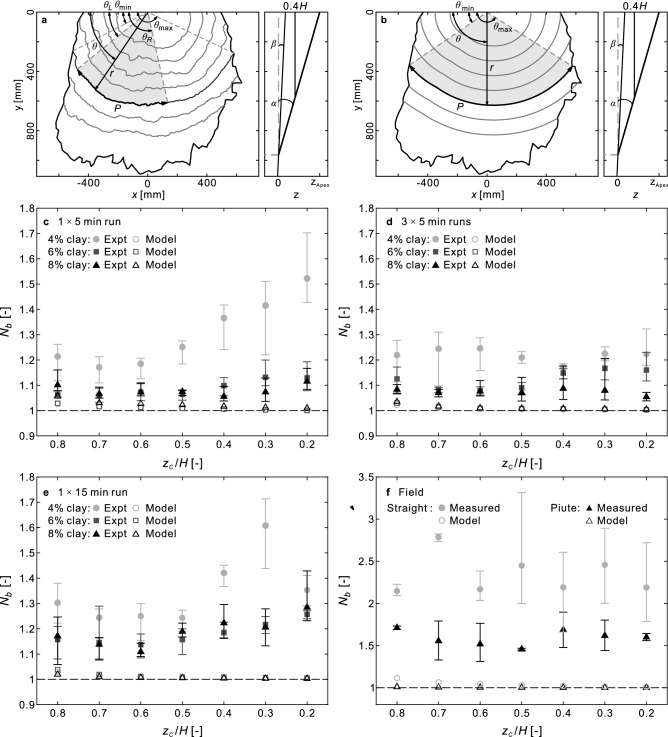
Complexity measurements. (**a,b**) Illustrative contours and complexity-related variables from (**a**) the experimental fan with 4% clay and 3 $\times$ 5 min runs. (**b**) A constant-slope modelled fan using the same fan apex position and median slope. (**a,b**) Left panels: top view showing each fan boundary (black outer line); contours of elevation for $z_c$ from 0.2*H*, to 0.8*H*, where $H=z_{apex}$ (gray lines). (**a,b**) right panels: center vertical cross-section (with distorted *z* scale). Black lines: bed plane of slope $\tan \beta$ and approximate fan surface of slope $\tan \alpha$; dashed line: horizontal plane of $z=0$; bold line: contour plane $z_c$ =0.4*H*. (**c**–**f**) complexity measurements from the experimental and field alluvial fans, modeled and measured, as noted in the upper left corners. open symbols: modelled fans; closed symbols: mean $N_b$ of the measured fans. Error bars represent range of $N_b$ values for 6 smaller sections of each contour of the measured fans and 2 smaller sections for each field fan.

To evaluate effects of boundary conditions, clay content, and intermittency on fan deposit surfaces and quantitatively evasive measures of channel numbers, we consider certain slope statistics (Fig. 3f) and a measure of *topographic complexity* (Fig. 4).

We consider three idealized fan morphologies to build our intuition of slope statistics: (1) single-slope morphologies represent alluvial fans to first order approximation^7,41,42^ which yields a single delta function for their slope distributions. (2) Near-single-slope morphologies with topographic depressions^15^ (e.g., slope variations) yield finite, narrow slope distributions. (3) Deposit morphologies edged by steep slopes, such as Bingham or Herschel–Bulkley fluids (historically used to approximate debris flows^43^) would^44^, give rise to a positively skewed slope distribution. All of our experimental fans exhibited some of each of these characteristics (Fig. 3f) with relatively narrow central (25% to 75%) boxes, skewed with a long positive tail.

We found that the experimental fans built with higher clay contents had lower median slopes and less skewness (representing smoother surfaces, fewer channels, and/or fewer lobes). For any particular clay content we found the fans built continuously (for 15 min) exhibited the larger median slope, wider slope distributions and larger skewness, (representing bumpier surfaces, more channels and/or more lobes). Similarly, we found straight Fan has a wider slope distribution than Piute Fan, and a similar median slope and skewness. Since Piute has significantly more silt than Straight but a similar clay contents, this is relatively consistent with our experimental results. To quantitatively assess local surface irregularities (for example, associated with channels and lobes), we draw from what many call the *granular physics* literature^45,46^ and calculate what is called a measure *complexity* ($N_b$). Here, we calculate the so-called complexity, deviations of a predicted curve (see “Methods” section). In this context we calculate the complexity ($N_b=P^2/(2A\Delta \theta )$) of several height contour lines within a fan segment of bounding angle $\Delta \theta$: *P* is the contour length; *A* is the area bounded by the fan apex and the contour line (Fig. 4, “Methods” section). We plot these results with corresponding contours for single-sloped model fans based on each experimental apex and apex slope. For the model fans, $N_b\sim 1$ and approaches 1 down-fan. For the experiments, $N_b$ increases with apparent surficial bumpiness (Fig. 3a–e). In this regard, we find complete consistency between $N_b$ and slope variability (Fig. 3f). That is, for lower clay content and longer continuous flow time, we obtain both the largest $N_b$ values and the widest slope distributions. $N_b$ also provides dependence on distance downslope. For the continuous 5 min. and 15 min. runs *only* using 4% clay (Fig. 4c,e) the relative complexity increase up-fan is statistically significant. For incrementally-deposited fans (Fig. 4d,f) there is no such identifiable down-slope trend. The field fans also do not appear to exhibit this consistent upstream complexity dependence, consistent with several flows built over time. As with our experimental fans, the field fan with the higher fine particle content (straight) has a higher $N_b$ value.

To summarize and more fully contextualize our findings, we first refer back to our remarks in the introduction regarding distinctions among fans built under different climatic and other boundary conditions. We noted in some cases debris flows explored the entire fan in a single event, and, in others, debris flows were restricted to a single channel or region of the fan. Our experimental fans—both incremental and continuous—developed under from experimental debris flows that built and explored their alluvial surface in each event, not unlike those reported in Taiwan Laonong River Valley, Fig. 1a)^4–6^. We hypothesize, then, that analysis of the Pu-tan-pu-nas fan, the Yu-Shui fan, and other fans that are relatively young or whose deposit is periodically washed away (e.g., as in the Taiwanese debris flow deposits) would reveal results more comparable to our experiments than the White Mountain Fans from our preliminary field work.

Perhaps surprisingly, our preliminary measurements and analysis from the White Mountain fans are consistent with our measures of fine-particle-dependence of slope statistics and topographic complexity. That is, in these field cases, the Straight fan, with its relatively small percentage of fine particles, exhibited a wider slope distribution and a higher topographic complexity than Piute Fan. We find this similarity with our experimental results surprising for two reasons. First, records indicate that their flow scale compared to that of the fan development is much shorter than those of our experiments, i.e., single flow events are restricted to relatively small regions of their fans. Second, the higher fines content on Piute (compared to Straight) pertains to silt rather than clay. Silt and clay particles are different in their settling velocities and relative interparticle interactions—clay particle-particle interactions are influenced by electromagnetic effects that can be tuned with different fluid solvents; silt particle-particle interactions are more typical of contact interactions of their larger counterparts. Thus we would initially suspect that they would affect the larger-scale flows in which they travel distinctly. On the other hand, at moderate concentrations in a Newtonian liquid, both give rise to a non-Newtonian fluid suspension/slurry. Man et al.^36,37^ demonstrated the presence of a non-Newtonian interstitial fluid whose rheology is analagous to that of granular suspensions is critical to the dynamics of compaction a particle-fluid mixture in many ways analagous to debris flows. It is not clear to us at present how differences in the rheology of different non-Newtonian interstitial fluids reflective of clay and/or silt particles in suspensions would give rise to specific flow, erosion, and avulsion behaviors of debris flows. We are working on these questions at different scales to derive a more complete understanding of this problem.

To wrap up, we suggest that the data and analyses we present in this paper provide a more complete foundation for the importance of fine particle content in debris flows that will ultimately be included in model frameworks for how fine particle content can influence debris-flow driven morphology on alluvial fans as well as their associated hazards. In addition to quantitative links between grain-size, intermittency, and channelizations, these results provide insights to design experiments more in-line with field-scale fan development. Additional tools will likely arise from investigations into micro-scale dynamics interparticle dynamics including the physical processes by which fine particles such as silt and clay affect larger-particle interactions and, cumulatively, influence macro-scale fan morphologies. New computational methodologies and other inspiring insights are becoming increasingly applicable from what we might consider the sibling field “granular physics” i.e., the study of how particle scale properties and interactions influence macroscopic behaviors in particle-flows and in particle-fluid-flows^37,47–49^. These and other advancements in particle-rich geomorphology and granular physics^50,51^ show exciting promise for a richer understanding, necessary for tackling complex problems in our natural and human infrastructure in this time of dramatically changing climates.

## Methods

### Field case analysis

For our field data we obtained: (1) documented lithology of the catchment areas; (2) the fine particle grain size distribution (2 mm and finer), often considered a substantial part of the muddy fluid or “matrix” of a debris flow, and (3) measures of slopes and complexity of channelization. We relied on a previously published USGS map^38^ for the lithology (Supplementary Fig. 1a and caption). We sampled the fine particle size distribution from regions shielded from non-debris flow events, such as rain-wash or fluvial transport. To do so, we sampled beneath large objects (e.g., boulders) in levee walls of what appeared to be the most recent flow event for that fan. To sample below the protective object, we scraped away the exposed material from the roughly vertical wall surface beneath it. Then we collected material from the region we exposed behind that for our measurements. For the Straight Fan, we took samples from the channel walls we associated with the historic debris flow recorded in 1918 based on the lighter coloration and previous documentation as in Ref.^52^. There is no recent, indeed no historical, debris flow on the Piute fan, so we sampled the sediment near the fan apex from within the top levee layer approximately 20 m above the bottom of what we judged to be the most recent channel based on apparent stream activity at its base. During the preliminary field visit, additional sample collection was not part of the scope, though we plan to do this during near future visits. We used sieves to measure the fractions of each grain size down to approximately 0.5 mm. For smaller sizes we used sedimentation (hydrometer) analysis^53^. For slopes and channelization, we used published Digital Elevation Model data^54^. We processed this for slopes and channel statistics, similar to the methodology for the experiments, described in the next section.

### Laboratory experiments

For our experimental mixtures, we used angular sand (specific gravity of 2.65, size distribution shown in Fig. 2b), clay (Kaolinite with specific gravity of 2.65), Polydiallyldimethylammonium chloride (PDADMAC), and water (Supplementary Table 4). We note two key details about the mixtures that differ from a typical debris flow to accommodate our experimental design: (1) We use a water content of approximately 44% by weight, compared with a more typical field-scale water content of 15–30% by weight^34^; we required this for instantaneously well mixing the sediments and fluids for sustained flows that mimics long-duration events in the field. (2) We used a small percentage of PDADMAC; we required this to flocculate clay particles, so that the clay would settle more quickly in our scaled-down experimental setting.

We prepared the materials to flow in the channel/basin experiment as follows. We filled the sediment feeder (center in Fig. 2a) and calibrated the output. Separately, we pre-mixed the clay, PDADMAC, and water with the designed ratio in the fluid mixing tank (center right of Fig. 2a) and pumped the fluid (clay–water mixture) into the head tank (top center in Fig. 2a). During the experiments we continuously circulate the fluid between the head tank and the mixing tank to assure we maintain a good mixture. To initiate an experiment, we simultaneously released the fluid and sand into a rotating funnel mixer from which they emerged well-mixed and flowed into the inclined channel (top-right center of Fig. 2a).

The channel was approximately 2 m long and 20 cm wide and for all experiments was inclined at a slope of 0.3. The sediment mixtures flowed continuously through the channel and into the top center of the basin (approximately 3 m wide by 5 m long and inclined at a slope of 0.118). To mimic boundary conditions in the field which are more porous than a typical basin boundary, we built a “permeable layer” (approximately 10 mm thick, covered by #200 mesh filter screen sheet, and filled with sand) onto which the sediment–fluid mixture flowed. Relatively quickly, fluid left the sediment layer through the permeable layer and, over time, the sediment deposit thickened into a cone-shaped deposit (e.g., Fig. 2c–e) with channel and lobe-like features as discussed in the text.

### Analysis techniques

For the analysis of the field and experimental data, we require high resolution digital elevation models (DEM) for each. We use published field data^54^ that provides data within a resolution of 1 m $\times$ 1 m, with uncertainty in z-direction from 5 to 35 cm. To reconstruct the topography of the experimental fans, we first took $\sim$ 600 images of the surface and of the 40 control points (Fig. 2a) from multiple viewpoints. Then, we used a commercial software (Agisoft Metashape) which uses a stereo analysis photogrammetry method to build a digital elevation model (DEM) and an orthophoto. By using the large number of control points and photographs for the scale of the fan we obtain a particularly high-precision DEM (2 mm $\times$ 2 mm grid size with uncertainty in z-direction of 1.2 mm) (provided as open access data D1–D9).

After obtaining high-accuracy DEMs for the experiments and field fans, we performed additional calculations to build slope maps and slope histograms to eliminate misleading data associated with individual grains. To do so, we first considered all the coordinates at which the 2 mm $\times$ 2 mm grid lines (for the experiments) or 1 m $\times$ 1 m (for the field) intersected with equal height contour spaced at 5 mm height intervals (experiments) or 1 m height intervals (for the field). Then for each such intersection point on a particular contour, we find the nearest intersection point on the neighboring contour and use the positions to calculate a local slope and temporarily assign it to the downstream neighbor. We do this for every point on every contour. Then we linear-interpolated the slopes onto the original 2 mm $\times$ 2 mm Cartesian grids (experiments) or 1 m $\times$ 1 m Cartesian grids (for the field) and obtained the slope maps as shown in the subfigures in Fig. 3 and Supplementary Fig. 4 and in Supplemental Data file D11–D20 (we provide data in open access 10.5281/zenodo.5721569). Since we are only concerned with the slope data on the fan, as indicated above, we used the orthophoto to define the fan boundary and cropped the slope data outside of the boundary. Finally, based on the cropped slope data, we calculated the distribution of the slope on the fan (present as boxplots in Fig. 3f), which provides one quantitative representation of all slope data.

To quantitatively assess the fan morphology in more detail, we turn back to individual contour lines from the slope plots detailed in Supplementary Fig. 4. As a representation of the morphology of the fan, we choose several equally spaced contour lines that span the majority of each fan. Then, we define a dimensionless number to examine the shape and bumpiness of each elevation contour of the fans. We define ($\theta _{\max }, \theta _{\min }$) as the maximum and minimum angles for which the contour is continuous from one edge of the fan to the next (Fig. 4a,b). We divide each contour into 6 overlapping sections which bounded by ($\theta _{L,k}, \theta _{R,k}$), where $\theta _{L,k}=\theta _{\min } + (k-1)(\theta _{\max }- \theta _{\min })/10$ and $\theta _{R,k}=\theta _{\max } - (6-k)(\theta _{\max }- \theta _{\min })/10$ for $k=1,2,...,6$. For each section, we calculate a complexity number as

1 $$\begin{aligned} N_b=\frac{P^2}{2A\Delta \theta }: \quad P=\int _{\theta _{L}}^{\theta _{R}} r\,d\theta , \quad A = \int _{\theta _{L}}^{\theta _{R}} \frac{1}{2}r^2 \,d\theta , \quad \Delta \theta =\int _{\theta _{L}}^{\theta _{R}} d\theta . \end{aligned}$$

In these equations, *r* references the polar coordinate of each vertex of a contour with the origin located at the apex of the fan. We note that in some cases, the contour is segmented because of missing in the field (e.g., Supplementary Fig. 4a), the value of the contour length *P*, the area *A*, and the opening angle $\Delta \theta$ are the summation of the integrations in each segment.

2 $$\begin{aligned} N_b=\frac{(\sum {P_i})^2}{2\sum {A_i}\sum {\Delta \theta _i}}; \quad P_i=\int _{\theta _{L,i}}^{\theta _{R,i}} r\,d\theta , \quad A_i = \int _{\theta _{L,i}}^{\theta _{R,i}} \frac{1}{2}r^2 \,d\theta , \quad \Delta \theta _i=\int _{\theta _{L,i}}^{\theta _{R,i}} d\theta . \end{aligned}$$

For each fan, we define the apex slope $\tan \alpha$ by the average deposit surface gradient from where 20 cm upstream the channel end to the channel end. Next, we compute a fan-specific $z=0$ where the plane of the bottom (permeable) boundary (slope $\tan \beta =0.118$) and an inclined plane defined by the fan apex height ($z_{Apex}$) and apex slope $\tan \alpha$ (see Fig. 4a, right column) intersect with each other. Then, we calculate $N_b$ for several contours whose elevation $z_c= 0.2H, 0.3H, ..., 0.8H$, where fan height $H=z_{Apex}$ based on the fan-specific $z=0$. For interpretation of the values of $N_b$, we note that for a circle segment, $N_b=1$, and both bumpiness and the distortion of curve shape will increase $N_b$. However, we also note that the shape of contours will be influenced by the inflow channel while closing to it. To separate the effects of the inflow channel from the real surface roughness, we propose a model fan that contains only a smooth and constant-slope surface for each actual fan. With the constant slope, an arbitrary contour of a modelled fan will always consist of three segments: a straight line segment of the length equal to the inflow channel width and two circular arcs (e.g. Fig. 4b). We calculate $N_b$ for corresponding contours on both actual fans and modelled fans (closed and open symbols respectively in Fig. 4c–f). Apart from the influence of the inflow channel, if the length of a curve segment has irregularities such as occurs on our fans for larger channel/lobe variations or greater numbers of channels or lobes, then $N_b$ increases (as *P* increases for relatively little change in *A*).

## Supplementary Information

Supplementary Information.

Supplementary Video S1.

Supplementary Video S2.

Supplementary Video S3.

Supplementary Video S4.

Supplementary Video S5.

Supplementary Video S6.

Supplementary Video S7.

Supplementary Video S8.

## Data Availability

The data, including the digital elevation models (DEM), the slope map data for 9 debris flow fan experiments and the slope map data for 2 field cases (the Straight Fan and Piute Fan in White Mountain, CA) are available at 10.5281/zenodo.5721569.
