# Supplementary material for: Influence of fine particle content in debris flows on alluvial fan morphology
Source: Sci Rep. 2022 Dec 16;12:21730. doi: 10.1038/s41598-022-24397-x (PMC9758158; doi:10.1038/s41598-022-24397-x)
Supplement: Supplementary file 1 — Supplementary Information. [file 41598_2022_24397_MOESM1_ESM.pdf]

Supplementary Information for “Influence of Fine Particle Content in Debris Flows on Alluvial Fan Morphology"

Tzu-Yin Kasha Chen[1,3], Chi-Yao Hung[2], Jared Mullenbach[3-5], and Kimberly Hill[3,4,*]

[1]National Taiwan University, Dept of Civil Engineering and Hydrotech Research Institute, Taipei, 10617, Taiwan
[2]National Chung Hsing University, Dept of Soil and Water Conservation, Taichung, 402, Taiwan
[3]University of Minnesota, Department of Civil, Environmental, and Geo- Engineering, Minneapolis, MN 55455, USA
[4]University of Minnesota, St. Anthony Falls Laboratory, Minneapolis, MN 55414, USA
[5]Wood Engineering, Reno, NV 89521,USA
[*]kmhill@umn.edu

1 Contents of this file

1.1 Supplementary Figures and Tables

In order of appearance in this Supplementary Document, the Figures and Tables are as follows:

- Supplementary Figure 1. Geological map and fine particle grain size distributions for the fields.
- Supplementary Table 1. Data from Straight and Piute Fans and catchment areas.
- Supplementary Table 2. GPS data for locations of the fine particle sampling in the field.
- Supplementary Figure 2. Plots of fine particle (d<2 mm) grain size distributions of the samples in the field.
- Supplementary Table 3. Fine particle (d<2 mm) grain size distributions of the samples in the field.
- Supplementary Table 4. Experimental flow compositions and discharges.
- Supplementary Table 5. Experiment sediment size distribution (plotted in Figure 2 inset) and mixture ratios.
- Supplementary Figure 3. Representative rate-dependent viscosity measurements of clay-water mixtures using a coaxial cylinder viscometer.
- Supplementary Table 6. Viscosity measurements of clay-water mixtures (Kaolinite-suspension fluids) for Supplementary Fig. 3.
- Supplementary Table 7. Summary of experiment runs, conditions, and associated video files/slope map.
- Supplementary Table 8. Legends, titles, and content of Supplementary Videos 1-8.
- Supplementary Table 9. Identifying information for the Supplementary Videos.
- Supplementary Figure 4. Elevation contours and slope map of the fans in the fields and experiments.
- Supplementary Figure 5. Slope distribution from surface of all fans in the fields and experiments.
- Supplementary Table 10. Slope distribution data from surface of all fans in the experiments.

1.2 Additional Supplementary Information (Files uploaded separately)

- Supplementary Videos 1-8. Videos from each debris fan experiment.

1.3 Open access Data sets

- Metadata to guide user in contents of open access data set.
- D1-D9 Digital elevation data (.tif files) for experiment topographies.
- D10-D20 slope maps (.tif files) for fields and experiments.

2 Supplementary Field Data

The first set of supplementary tables and figures show information about particle properties and grain size distributions in the field sites.

Supplementary Figure 1 shows a section of a field geological map developed by Crowder et al. (1972)[1]. The section was chosen to provide information on the dominant lithology of the catchment areas of Straight Creek and Paiute Creek. Data from Straight and Piute fans and catchment areas are presented in Supplementary Table 1. Supplementary Table 1 provides heights and *effective* slopes of the catchment areas of the two fans from google earth data. What we refer to as *effective slopes*, was first proposed as a helpful measure by Melton (1965)[2] and is a representative slope of the catchment area, i.e., $R = H/\sqrt{A}$; H and A represent the maximum vertical relief and the planview area, respectively.

Supplementary Table 2 denotes the GPS coordinates of five sample locations of the fans, which are also indicated in Figure 1b of the main text. Supplementary Figure 2 shows the measured grain size distribution obtained on these sample locations. The grain size distribution data are included in Supplementary Table 3.

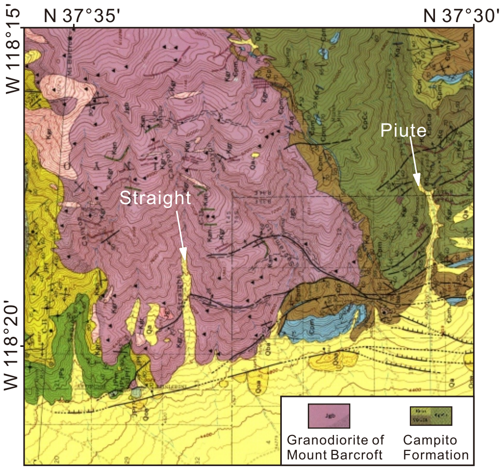

Figure 1. Field geological map[1]. The catchment material of Straight Creek and fan is primarily granodiorite, a relatively hard (∼225 MPa) coarse-grained intrusive igneous rock comprised of quartz and plagioclase feldspar. In contrast, the catchment material of Piute Creek primarily consists of hornfels, contact metamorphic rock that is considerably less hard (∼5.8 MPa). Additionally, at the base of the Piute catchment, adjacent to the apex of the fan, the material is that of the poleta formation, mostly sedimentary rock, silt and limestone[1,3].

Table 1. Data from Straight and Piute Fans and catchment areas. S_i refers to the slope for which $i\%$ of the slopes are smaller.

Fan Name		Straight	Piute
Catchment	Max height [m]	3747	3836
	Min height [m]	1577	1635
	Drainage area [km^2]	6.29	15.08
	Meltons number [-]	0.87	0.57
Fan	Fan area [km^2]	3.12	3.79
	S_1	0.0411	0.0778
	S_5	0.0593	0.1085
	S_{25}	0.1012	0.1301
	S_{50}	0.1440	0.1449
	S_{75}	0.1943	0.1640
	S_{95}	0.3197	0.2337
	S_{99}	0.4864	0.3820

Table 2. GPS data for locations of the fine particle sampling in the field.

	UTM zone 11		
Fan Name	Easting (m)	Northing (m)	Elevation (m)
Piute	382956	4152326	1565
Straight 1	381728	4157467	1548
Straight 2	381512	4157255	1503
Straight 3	381328	4157184	1470
Straight 4	380919	4157066	1411

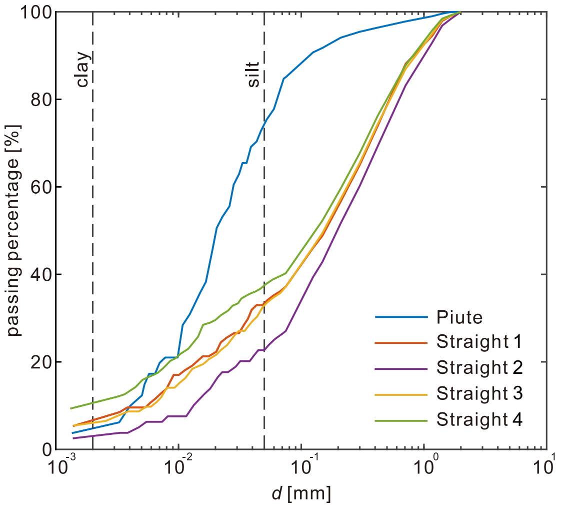

Figure 2. Fine particle (d < 2 mm) grain size distribution for Straight and Piute in the White Mountains (California, USA). Based on our measurements, the matrix particles of the Straight fan deposit are more than 60% sand while the finer grains on the Piute grains were more than 75% silt or finer.

Table 3. Sediment size distribution for the field (only showing the part of sand/silt/clay (d<2mm) (Information for sampling location see Supplementary Table 2).

Piute		Straight 1		Straight 2		Straight 3		Straight 4	
grain size (mm)	passing rate (%)	grain size (mm)	passing rate (%)	grain size (mm)	passing rate (%)	grain size (mm)	passing rate (%)	grain size (mm)	passing rate (%)
2.00000	100.0	2.00000	100.0	2.00000	100.0	2.00000	100.0	2.00000	100.0
1.65000	100.0	1.65000	99.0	1.65000	98.3	1.65000	99.0	1.65000	99.2
1.41000	99.6	1.41000	97.9	1.41000	96.8	1.41000	98.1	1.41000	98.4
1.16800	99.0	1.16800	94.5	1.16800	92.8	1.16800	94.9	1.16800	95.8
0.71000	97.8	0.71000	88.1	0.71000	82.7	0.71000	86.9	0.71000	87.8
0.41700	96.3	0.41700	73.9	0.41700	68.3	0.41700	74.1	0.41700	76.2
0.29700	95.4	0.29700	65.0	0.29700	58.9	0.29700	65.2	0.29700	67.7
0.21000	94.0	0.21000	56.7	0.21000	50.1	0.21000	57.0	0.21000	59.6
0.14900	91.7	0.14900	48.8	0.14900	41.0	0.14900	49.1	0.14900	52.3
0.12500	90.6	0.12500	46.1	0.12500	37.4	0.12500	45.9	0.12500	49.2
0.07500	84.9	0.07500	37.2	0.07500	24.7	0.07500	36.7	0.07500	40.3
0.07197	83.7	0.06766	36.1	0.07058	24.2	0.06794	34.7	0.07145	39.9
0.05990	78.7	0.05882	35.1	0.06113	23.0	0.05930	33.6	0.05894	38.9
0.05212	76.2	0.05261	34.0	0.05487	21.9	0.05304	32.6	0.05155	37.8
0.04705	73.7	0.04839	33.0	0.05009	20.7	0.04860	31.5	0.04634	36.7
0.04334	71.2	0.04328	33.0	0.04480	20.7	0.04380	29.4	0.04271	36.1
0.03894	69.9	0.03951	31.9	0.04118	19.6	0.03999	28.4	0.03820	35.6
0.03587	66.2	0.03672	29.8	0.03813	18.4	0.03716	27.3	0.03521	35.0
0.03321	66.2	0.03461	28.7	0.03566	18.4	0.03476	26.3	0.03260	34.5
0.03120	63.7	0.03118	26.6	0.03190	18.4	0.03109	26.3	0.03064	33.4
0.02814	61.2	0.02846	26.6	0.02932	17.3	0.02860	25.2	0.02766	32.8
0.02602	56.2	0.02465	25.5	0.02539	16.1	0.02495	23.1	0.02548	31.7
0.02272	53.7	0.02213	24.5	0.02271	16.1	0.02231	22.1	0.02216	30.6
0.02049	51.2	0.02035	22.3	0.02080	15.0	0.02044	21.0	0.02000	29.5
0.01893	46.2	0.01762	21.3	0.01814	12.7	0.01770	20.0	0.01826	29.0
0.01671	38.7	0.01576	21.3	0.01622	11.5	0.01595	18.9	0.01588	28.5
0.01500	36.2	0.01296	19.1	0.01333	9.2	0.01302	17.9	0.01445	25.7
0.01234	31.2	0.01126	18.1	0.01158	6.9	0.01136	15.8	0.01204	23.0
0.01081	28.7	0.01007	17.0	0.01036	6.9	0.01020	14.7	0.01047	21.9
0.00984	21.2	0.00914	17.0	0.00946	6.9	0.00931	13.7	0.00944	20.8
0.00898	21.2	0.00797	13.8	0.00819	6.9	0.00806	13.7	0.00862	20.2
0.00778	21.2	0.00718	12.8	0.00737	5.8	0.00726	11.6	0.00755	18.6
0.00701	20.0	0.00656	11.7	0.00673	5.8	0.00667	10.5	0.00681	17.5
0.00644	17.5	0.00588	10.6	0.00602	5.8	0.00597	9.5	0.00621	17.0
0.00576	17.5	0.00537	9.6	0.00550	5.8	0.00547	9.5	0.00558	16.4
0.00528	15.0	0.00465	9.6	0.00476	4.6	0.00477	8.4	0.00509	15.9
0.00504	12.5	0.00382	9.6	0.00386	3.5	0.00389	8.4	0.00448	14.2
0.00401	10.0	0.00331	8.5	0.00335	3.5	0.00256	6.3	0.00365	12.6
0.00328	6.2	0.00138	5.3	0.00138	2.3	0.00139	5.3	0.00318	12.0
0.00135	3.7							0.00131	9.3

3 Supplementary Laboratory Data

The second set of supplementary tables and figures contain information about the material properties and conditions adopted in the laboratory experiments.

3.1 Experimental Mixture Contents

We used three different mixtures varying predominantly with clay content which, from low to high clay content, we refer to as: (1) debris floods, (2) runny debris flows, and (3) viscous debris flows, respectively. Supplementary Table 4 shows the flow compositions and relative discharges of the sand, clay, water, and flocculant (PDAMAC) for all of the experimental runs runs. Supplementary Table 5 shows the grain size distributions (plotted in the inset of Figure 2 of the main text). The specific mixtures were chosen based on their distinct behaviors in small-scale bench-top studies performed for a wider range of sediment mixtures (in preparation).

Supplementary Figure 3 and Supplementary Table 6 shows the steady-state "rheology" measurements from a Brookfield DV-II+ viscometer. Specifically, we report rotation rate-dependent stresses and machine-output viscosities of two clay-water mixtures used in our debris flow-experiments (12 % and 16% clay content in the interstitial fluids). We note that the Brookfield DV-II+ viscometer calculates viscosity based on an assumption of Newtonian fluid, thus cannot be representative of the actual flow-dependence as the local shear rate varies with distance from the spindle. We also present the viscosity calculated from the formula in de Haas et al. (2015)[4] as comparison in Supplementary Table 6.

Table 4. Experimental flow compositions and discharges.

Label	Exp run #'s	Sand (w%)	Clay (w%)	PDADMAC (w%)	Water (w%)
		Sand discharge (g/s)	Fluid discharge (g/s)		
Debris flood	R1 - R4	50	4	0.004	45.996
		100	100		
Runny debris flow	R5 - R8	52	6	0.006	41.994
		100	92		
Viscous debris flow	R9 - R12	48	8	0.008	43.992
		100	108		

Table 5. Experiment sediment size distribution (plotted in Figure 2 inset) and mixture ratios.

	Debris flood	Runny debris flow	Viscous debris flow
Sieve size (mm)	sediment passing rate (%)		
4.75	100	100	100
2.36	92.3	91.9	91.7
1.18	76	74.9	74.1
0.6	53.7	51.6	50
0.3	28.9	25.6	23.1
0.15	16	12.1	9.3
0.075	14.8	10.9	8.0
clay:fluid ratio	0.08	0.12	0.16
clay:sediment ratio	0.074	0.10	0.14
clay:mixture ratio	0.04	0.06	0.08

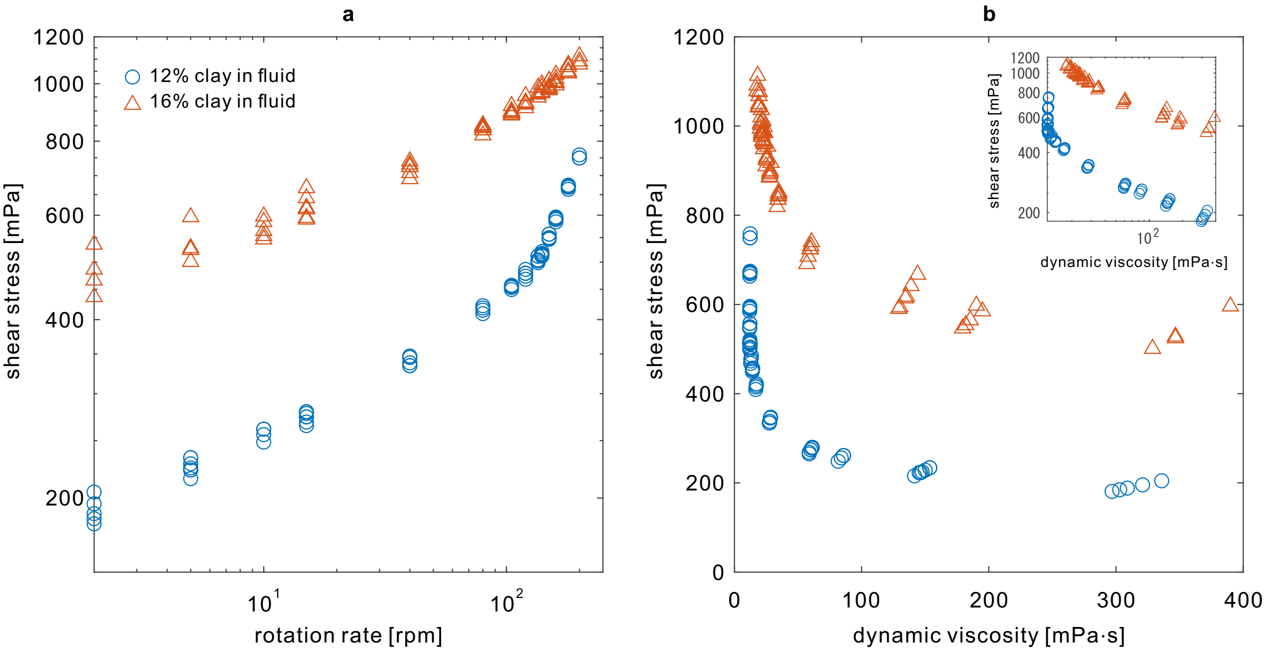

Figure 3. Representative viscosity measurements of clay-water mixtures using a coaxial cylinder viscometer (Brookfield). The spindle radius = 0.95 cm; fill height = 6.5 cm, and fluid container radius = 1.375 cm. (a) Shear stress versus rotation rate. (b) Shear stress versus dynamic viscosity, output from the viscometer. Inset: plot in log-linear scale. Measurement data are presented in Supplementary Table 6.

Table 6. Viscosity measurements of clay-water mixtures (Kaolinite-suspension fluids) for Supplementary Fig. 3 using a coaxial cylinder rheometer with a spindle radius of 0.95 cm and height of 6.5 cm, and a fluid container radius of 1.375 cm.

12 w% clay in fluid			16 w% clay in fluid		
rotation rate [rpm]	viscocity [mPa-s]	shear stress [mPa]	rotation rate [rpm]	viscocity [mPa-s]	shear stress [mPa]
2	296.9	181.0	2	716.8	436.8
5	141.6	215.7	5	328.7	500.8
10	81.6	248.6	10	181.8	553.8
15	60	274.2	10	179.4	546.5
15	58.8	265.0	15	130	594.0
40	27.7	334.5	40	58	707.4
80	16.8	409.4	40	56.9	690.9
105	14.1	449.6	80	33.6	818.8
120	13.1	478.9	105	27.7	884.6
120	12.8	467.9	120	25.2	923.0
135	12.1	499.0	120	24.9	910.2
140	12	511.8	135	23.1	948.6
140	12	513.6	140	22.6	965.1
150	12	550.2	150	21.6	988.8
160	12	584.9	150	21.4	976.0
160	12.1	594.0	160	20.6	1003.5
180	12.2	670.8	180	19	1043.7
200	12.4	758.5	200	17.9	1091.2
200	12.3	749.4	200	17.7	1078.4
180	12.3	674.5	180	19	1041.8
160	12.2	595.9	160	20.6	1003.5
160	12.1	592.2	150	21.6	985.2
150	12.2	557.5	150	21.4	979.7
140	12.2	520.9	140	22.7	966.9
135	12.4	511.8	135	23.3	959.6
135	12.2	502.6	120	25.4	930.3
120	13.1	478.9	105	28	895.6
105	14.3	456.9	105	27.7	886.5
80	17.3	422.2	80	34.3	835.3
40	28.5	347.3	40	59.4	723.8
15	61.2	279.7	15	134.4	614.1
10	85.8	261.4	15	129.2	590.4
5	153.6	234.0	10	185.4	564.8
5	146.4	223.0	5	346.7	524.6
2	320.9	195.6	2	797.8	486.2
5	150	228.5	2	767.8	466.1
5	147.6	224.8	5	346.7	528.2
10	84	255.9	10	190.2	597.7
15	61.2	279.7	15	135.2	617.8
15	58.8	268.7	40	60	731.1
15	60	274.2	80	34.9	851.7
40	28.3	345.5	80	34.8	848.1
40	27.4	334.5	105	28.7	917.6
80	17	414.9	120	26.1	954.1
105	14.3	456.9	135	23.9	985.2
105	14.2	455.1	140	23.4	998.0
120	13.3	486.2	140	23.1	985.2
135	12.4	511.8	150	22.1	1010.8
135	12.2	502.6	150	22.2	1012.6
140	12.1	515.4	160	21.3	1036.4
150	12.2	557.5	160	20.9	1019.9
150	12	546.5	180	19.4	1067.4
160	12.1	590.4	200	18.3	1113.1
180	12.1	663.5	180	19.6	1076.6
180	12.2	669.0	180	19.1	1049.2
180	12.2	669.0	160	20.7	1007.1
200	12.3	749.4	160	20.4	994.3
180	12.3	672.6	150	21.5	983.4
180	12.3	672.6	140	22.7	968.7

Continued Supplementary Table 6.

12 w% clay in fluid			16 w% clay in fluid		
rotation rate [rpm]	viscocity [mPa-s]	shear stress [mPa]	rotation rate [rpm]	viscocity [mPa-s]	shear stress [mPa]
160	12.1	590.4	135	23.4	961.4
150	12	548.3	135	23.1	948.6
140	12.1	517.3	120	25.3	926.7
140	12	513.6	105	28.1	899.3
135	12.3	504.5	105	27.9	892.0
120	13.1	478.9	80	34.6	844.4
120	12.9	473.4	80	34.6	842.6
105	14.2	453.3	80	34.3	835.3
80	17.2	418.6	40	60.6	740.3
40	28.3	345.5	40	59.7	723.8
40	27.8	338.1	15	144	667.1
15	60.8	277.8	15	138.8	641.6
10	85.8	261.4	10	195	584.9
5	153.6	234.0	5	389.9	595.9
2	335.9	204.7	2	890	535.5
2	302.9	184.6			
2	308.9	188.3			
5	145.2	223.0			
Calculated viscosity from formula in de Haas et al. (2015)[4]: $\mu = (1 + 2.5v_{fine} + 10.05v_{fine}^2 + 0.00273\exp(16.6v_{fines}))\mu_w$, where v_{fines} is the volume fraction of fine particle in fluid and μ_w represents the dynamic viscosity of pure water $\mu_w = 1.002$ mPa-s					
1.15 mPa-s			1.22 mPa-s		

3.2 Experimental Run Conditions

Supplementary Table 7 shows the experimental conditions and associated files of all the experiment runs.

Table 7. Experiment runs: durations, flow types, and associated video files/slope maps.

Run #	basal boundary	duration	continuous (c) or incremental (i)	flow type	% clay	video file name	Slope map in Supplementary Fig. 4
R1	permeable bed	5 min	i	debris flood	4	V4 & V7	Yes, Fig. S4c
R2	top of run 1 deposit	5 min	i	debris flood	4	V4 & V7	No
R3	top of run 2 deposit	5 min	i	debris flood	4	V4 & V7	Yes, Fig. S4f
R4	permeable bed	15 min	c	debris flood	4	V3	Yes, Fig. S4i
R5	permeable bed	5 min	i	runny debris flow	6	V2	Yes, Fig. S4d
R6	top of run 5 deposit	5 min	i	runny debris flow	6	V2	No
R7	top of run 6 deposit	5 min	i	runny debris flow	6	V2	Yes, Fig. S4g
R8	permeable bed	15 min	c	runny debris flow	6	V1	Yes, Fig. S4j
R9	permeable bed	5 min	i	viscous debris flow	8	V6 & V8	Yes, Fig. S4e
R10	top of run 9 deposit	5 min	i	viscous debris flow	8	V6 & V8	No
R11	top of run 10 deposit	5 min	i	viscous debris flow	8	V6 & V8	Yes, Fig. S4h
R12	permeable bed	15 min	c	viscous debris flow	8	V5	Yes, Fig. S4k

3.3 Supplementary Videos

We include videos from many of the experiments in the supplementary materials to help the reader understand the evolving nature of the flow and deposits, particularly as the boundary conditions change from a porous base to a erodible deposit. We note the terminology we use referring to the type of the flow (i.e., debris flood, runny debris flow, and viscous debris flow). These are common nomenclatures in the literature but often have overlapping and even non-consistent uses. Initially, the "debris flow nature" of the dynamics are difficult to determine in all cases as the flows appear wide and runny. Nevertheless they show feature common to debris flows such as the largest particles (i.e., sand in this case) suspended on top of the flow in the channel. All flows are wide until an initial deposit is established. Then the flow behavior on the fan evolves from the early sheet flow behavior to the later channelized behavior. For all cases, this channelization is noticeable in the films. Other properties common to debris flows evidenced in these videos are lobe formation. Video contents and mixtures used in each video are listed in Tables 8 and 9, respectively.

From the Supplementary Video V1, we can see clear channelization at 0:22, 0:37, 0:44, 0:58, 1:12, 1:30, and 1:49, deposition of lobes at 0:33, 0:55, 1:02, 1:42, 1:54, back fill at 1:01-1:08, and gradual avulsion at 0:43, 1:10, 1:18, 1:29, and 1:53. From the Supplementary Video V2, we can see clear channelization at 1:12 and 1:42, deposition of lobes at 1:27 and 1:44, and gradual avulsion at 1:16. From the Supplementary Video V3, we can see clear channelization at 0:12, 0:15, 0:25, 0:27, 0:33, 0:55, 1:21 and 1:38, deposition of lobes at 0.55, 1:01, and 1:37, and gradual avulsion at 0:14, 0:17, 0:35, 0:56, 1:05, 1:34, and 1:44. From the Supplementary Video V4, we can see clear channelization at 0:20, 0:28, 0:35, 0:39, 0:56, 1:14, 1:39, and 1:48, deposition of lobes at 0.35 and 1:10, and gradual avulsion at 0:23, 0:32, 1:02, 1:21, 1:42, 1:56 and 2:04.

Table 8. Legends, Titles, and Contents for Supplementary Videos 1-8

Legend	Title	content
V1	V1C615MinsSideViewacc8640p.mov	Video record (side view) of the experiment process of a continuous (15 minute) flow with 6% clay mixture.
V2	V2C63x5MinsSideViewacc8640p.mov	Video record (side view) of the experiment process of three successive (5 minute) flow with 6% clay mixture.
V3	V3C415minsSideViewacc8640p.mov	Video record (side view) of the experiment process of a continuous (15 minute) flow with 4% clay mixture.
V4	V4C43x5minsSideViewacc8640p.mov	Video record (side view) of the experiment process of three successive (5 minute) flow with 4% clay mixture.
V5	V5C815minsSideViewacc8640p.mov	Video record (side view) of the experiment process of a continuous (15 minute) flow with 8% clay mixture.
V6	V6C83x5minsSideViewacc8640p.mov	Video record (side view) of the experiment process of three successive (5 minute) flow with 8% clay mixture.
V7	V7C43x5minsFrontViewacc8640p.mov	Video record (front view) of the experiment process of three successive (5 minute) flow with 4% clay mixture.
V8	V8C83x5minsFrontViewacc8640p.mov	Video record (front view) of the experiment process of three successive (5 minute) flow with 8% clay mixture.

Table 9. Supplementary video contents.

Video legend	Clay content (w%)	Water+ Flocculant content (w%)	Sand content (w%)	Sand discharge (g/s)	Fluid discharge (g/s)	Flow event time scale (min)	View direction
V1	6	42	52	100	92	1 x 15	side view
V2	6	42	52	100	92	3 x 5	side view
V3	4	46	50	100	100	1 x 15	side view
V4	4	46	50	100	100	3 x 5	side view
V5	8	44	48	100	108	1 x 15	side view
V6	8	44	48	100	108	3 x 5	side view
V7	4	46	50	100	100	3 x 5	front view
V8	8	44	48	100	108	3 x 5	front view

4 Field and Laboratory Fan Topographies and Complexities

In this last section, we provide analysis to supplement that in the main paper. Supplementary Figure 4 presents the topographies of the nine experimental fan surfaces that are most easily comparable (same total time of deposition or initiated on a cleaned permeable bed) and of two field fans. Supplementary Figure 4 presents elevations by contours (black lines) and local slope by colors (white to red). Supplementary Figure 5 supplements the box plots presented in the main text with full slope distributions, while Supplementary Table 10 presents the numerical values of the slope distributions on the fan surfaces from the box plots.

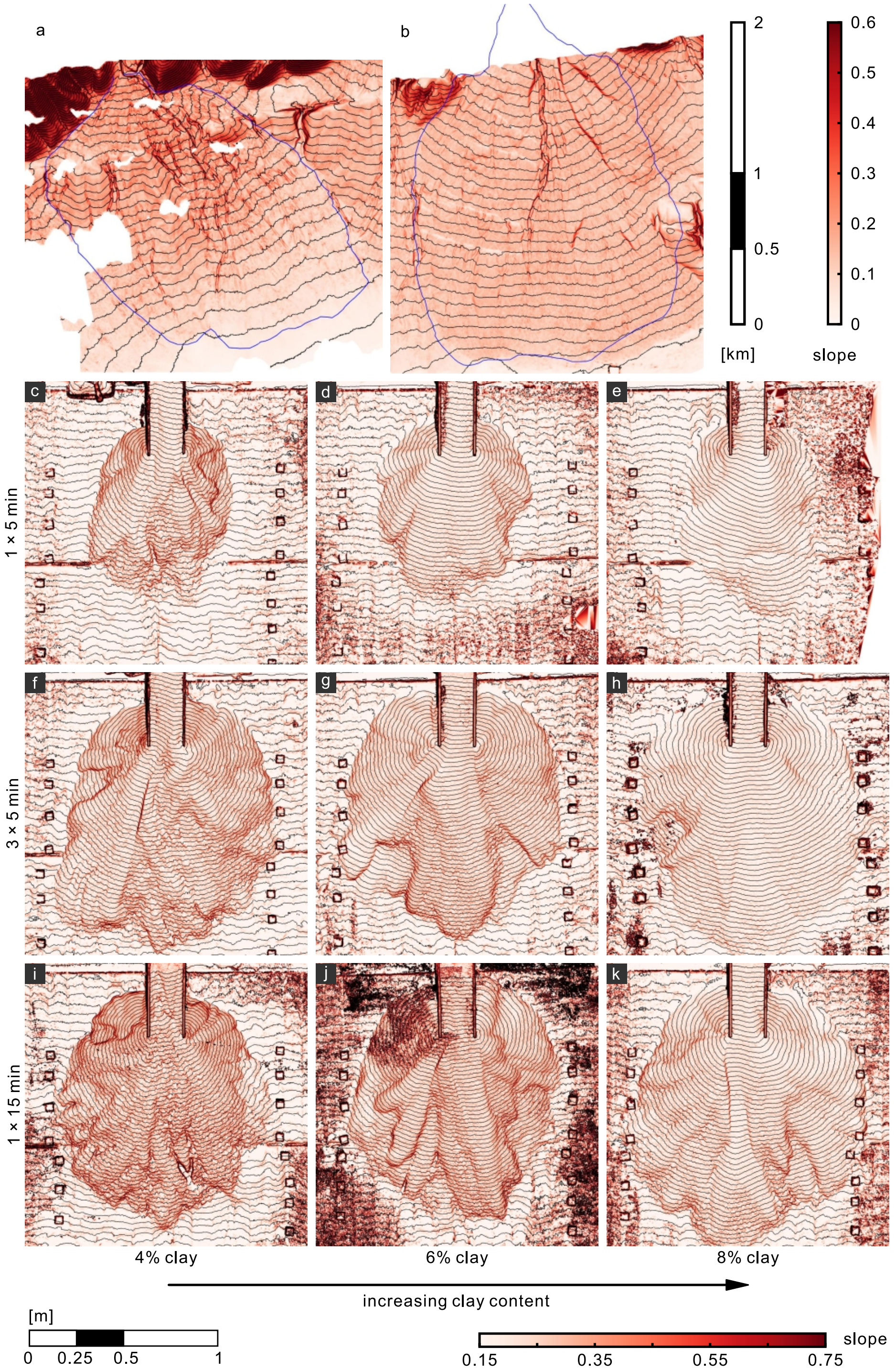

Figure 4. Elevation contours and slope map of the fans in the fields and experiments (Fig. 3 from the text extended to include all fans we discussed in this paper). **a**, Straight Fan. **b**, Piute Fan. **c-e**, 1 x 5 min run. **f-h**, 3 x 5 min runs. **i-k**, 1 x 15 min run. **c,f,i**, 4% clay. **d,g,j**, 6% clay. **e,h,k**, 8% clay.

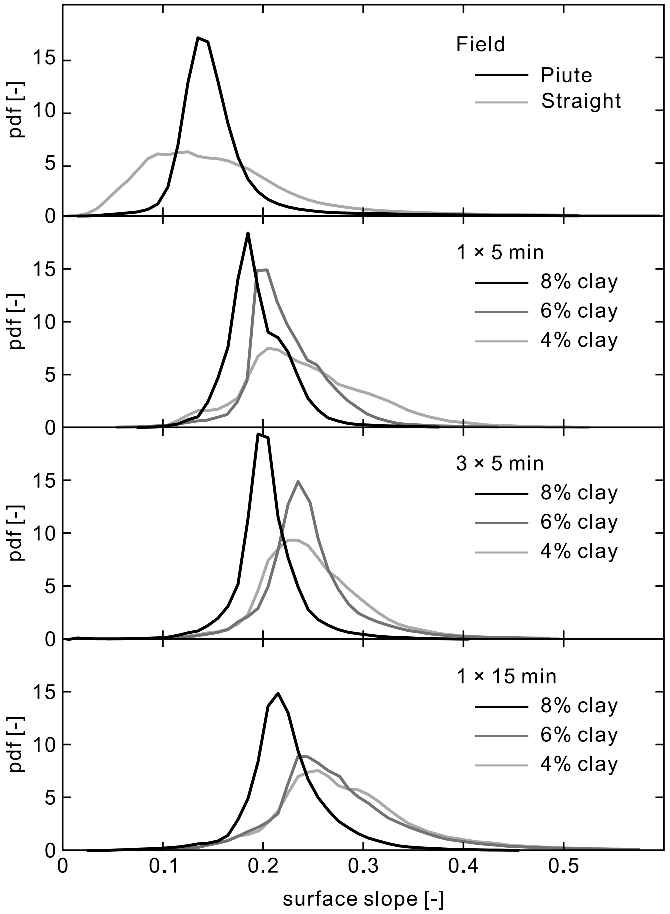

Figure 5. Slope distribution from surface of all fans in the field and experiments.

Table 10. Slope distribution data from surface of all fans in the experiments. S_i refers to the slope for which i% of the slopes are smaller.

Run #	R1	R5	R9	R3	R7	R11	R4	R8	R12
Duration	1 x 5 min			3 x 5 min			1 x 15 min		
Clay content [%]	4	6	8	4	6	8	4	6	8
S_1	0.1186	0.1268	0.1270	0.1281	0.1431	0.1032	0.1458	0.1468	0.1104
S_5	0.1465	0.1728	0.1508	0.1785	0.1831	0.1537	0.1857	0.1858	0.1675
S_{25}	0.2013	0.1994	0.1764	0.2163	0.2221	0.1892	0.2384	0.2354	0.2027
S_{50}	0.2366	0.2176	0.1904	0.2432	0.2398	0.2020	0.2729	0.2647	0.2198
S_{75}	0.2864	0.2466	0.2140	0.2784	0.2632	0.2186	0.3186	0.3073	0.2431
S_{95}	0.3724	0.2979	0.2516	0.3508	0.3343	0.2690	0.4263	0.4108	0.3013
S_{99}	0.4955	0.3686	0.3294	0.4456	0.4361	0.3844	0.5834	0.5802	0.3778

References

1. Crowder, D. F. & Sheridan, M. F. Geologic map of the White Mountain Peak quadrangle, Mono County, California. Geol. Quad. Map GQ-1012, U.S. Geol. Survey (1972).
2. Melton, M. A. Debris-covered hillslopes of the southern arizona desert: consideration of their stability and sediment contribution. *The J. Geol.* **73**, 715–729 (1965).
3. McKee, E. H. & Gangloff, R. A. Stratigraphic distribution of archaeocyathids in the Silver Peak Range and the White and Inyo Mountains, western Nevada and eastern California. *J. Paleontol.* 716–726 (1969).

4. De Haas, T., Braat, L., Leuven, J. R., Lokhorst, I. R. & Kleinhans, M. G. Effects of debris flow composition on runout, depositional mechanisms, and deposit morphology in laboratory experiments. *J. Geophys. Res. Earth Surf.* **120**, 1949–1972 (2015).
